# Exercise‐induced elevations in cerebral blood velocity are greater in running compared to cycling at higher intensities

**DOI:** 10.14814/phy2.14539

**Published:** 2020-08-12

**Authors:** Rhodri J. Furlong, Samuel R. Weaver, Rory Sutherland, Claire V. Burley, Gabriella M. Imi, Rebekah A. I. Lucas, Samuel J. E. Lucas

**Affiliations:** ^1^ School of Sport, Exercise and Rehabilitation Sciences College of Life and Environmental Sciences University of Birmingham Birmingham UK; ^2^ Centre for Human Brain Health University of Birmingham Birmingham UK; ^3^ Dementia Centre for Research Collaboration School of Psychiatry University of New South Wales Sydney Australia

**Keywords:** cerebral blood flow, cerebrovascular adaptation, exercise modality, high‐intensity exercise

## Abstract

The optimal exercise intensity and modality for maximizing cerebral blood flow (CBF) and hence potential exposure to positive, hemodynamically derived cerebral adaptations is yet to be fully determined. This study compared CBF velocity responses between running and cycling across a range of exercise intensities. Twenty‐six participants (12 females; age: 26 ± 8 years) completed four exercise sessions; two mode‐specific maximal oxygen consumption (VO_2max_) tests, followed by (order randomized) two incremental exercise protocols (3‐min stages at 35%, 50%, 65%, 80%, 95% VO_2max_). Continuous measures of middle cerebral artery velocity (MCAv), oxygen consumption, end‐tidal CO_2_ (P_ET_CO_2_), and heart rate were obtained. Modality‐specific MCAv changes were observed for the whole group (interaction effect: *p* = .01). Exercise‐induced increases in MCAv_mean_ during cycling followed an inverted‐U pattern, peaking at 65% VO_2max_ (Δ12 ± 7 cm/s from rest), whereas MCAv_mean_ during running increased linearly up to 95% VO_2max_ (change from rest: Δ12 ± 13 vs. Δ7 ± 8 cm/s for running vs. cycling at 95% VO_2max_; *p* = .01). In contrast, both modalities had an inverted‐U pattern for P_ET_CO_2_ changes, although peaked at different intensities (running: 50% VO_2max_, Δ6 ± 2 mmHg; cycling: 65% VO_2max_, Δ7 ± 2 mmHg; interaction effect: *p* = .01). Further subgroup analysis revealed that the running‐specific linear MCAv_mean_ response was fitness dependent (Fitness*modality*intensity interaction effect: *p* = .04). Above 65% VO_2max_, fitter participants (*n* = 16; male > 45 mL/min/kg and female > 40 mL/min/kg) increased MCAv_mean_ up to 95% VO_2max_, whereas in unfit participants (*n* = 7, male < mL/min/kg and female < 35 mL/min/kg) MCAv_mean_ returned toward resting values. Findings demonstrate that modality‐ and fitness‐specific profiles for MCAv_mean_ are seen at exercise intensities exceeding 65% VO_2max_.

## Graphical abstract

This study compared cerebral blood flow (CBF) responses between running and cycling across a range of exercise intensities. Our findings demonstrate that exercise‐induced increases in CBF (as indexed from Doppler‐based measures of middle cerebral artery velocity, MCAv) during incremental running and cycling differ at higher exercise intensities, with the pattern of the MCAv response during running for fitter individuals dissociating from the regulatory influence of PCO_2_ at near maximal intensity (95% VO_2max_). Thus, modality‐specific differences in beat‐to‐beat flow patterns may alter CBF regulation processes and affect the complex integration of factors regulating CBF during exercise.

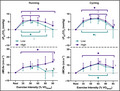

## 1 INTRODUCTION

Effective regulation of blood to and within the brain is vital for optimal brain function. Regular exercise and higher cardiorespiratory fitness has been positively linked with improved cerebral blood flow (CBF) and its regulation (including cerebrovascular resistance, compliance and reactivity (Bailey et al., 2013; Zimmerman et al., 2014), shown to offset the natural age‐related decline in CBF (Ainslie et al., 2008), and lower the risk of stroke (Lee, Folsom, & Blair, 2003; Pandey et al., 2016) and neurodegenerative diseases (e.g., dementia (Larson et al., 2006)). However, the mechanisms driving the vasoprotective benefits of exercise remain to be established, meaning that the effectiveness of various exercise parameters, such as mode, intensity, frequency, and duration, are not yet fully understood (Lucas, Cotter, Brassard, & Bailey, 2015). Improved understanding of how different exercise strategies induce the stimulus for positive cerebrovascular adaptation will allow a more targeted approach to optimize brain vascular health through exercise (Burley, Bailey, Marley, & Lucas, 2016).

Exercise‐induced increases in blood flow are a key component of the mechanism driving vascular adaptation in response to exercise (Lu & Kassab, 2011; Tinken et al., 2010). Specifically, changes in the hemodynamic forces acting on the vessel wall, due to elevated friction (shear stress) and distension (cyclic strain) as flow increases, stimulate the release of nitric oxide (NO) and a cascade of signaling events mediating positive structural and functional vascular adaptation (reviewed in (Green, Hopman, Padilla, Laughlin, & Thijssen, 2017)). Similarly, shear stress and cyclic strain have been identified as potential drivers of vascular change within the cerebrovasculature (Bolduc, Thorin‐Trescases, & Thorin, 2013; Padilla et al., 2011). However, to date the basis of this assertion has primarily come from animal‐based and cell culture studies, or inferred from the peripheral vasculature of humans. Given this, and the knowledge that the regulation of the cerebrovascular is distinct from the peripheral vasculature (Ogoh & Ainslie, 2010), the effect of exercise on CBF requires specific investigation. Furthermore, the regulation of CBF during exercise is complex and the interaction of key regulatory variables (e.g., arterial partial pressure of carbon dioxide, blood pressure, cardiac output) may differ with different exercise types (Smith & Ainslie, 2017). Thus, it is unclear how we might best optimize exercise intensity and modality in order to maximize CBF and subsequent hemodynamic stimuli for cerebrovascular adaptation.

The typical pattern of CBF reported in the literature describes an inverted‐U response with increasing exercise intensities, where CBF peaks at ~65% of maximal aerobic capacity (i.e., VO_2max_) before returning toward resting values at higher exercise intensities—with this profile coupled to comparable changes in end‐tidal carbon dioxide (reviewed in (Smith & Ainslie, 2017)). However, the vast majority of studies to date have used cycling to illustrate this relationship (e.g., Brugniaux, Marley, Hodson, New, & Bailey, 2014; Hellstrom, Fischer‐Colbrie, Wahlgren, & Jogestrand, 1996; Moraine et al., 1993)), in spite of variation in the CBF‐exercise intensity relationship in the few studies that have used alternative exercise modalities (e.g., rowing (Faull, Cotter, & Lucas, 2015; Pott et al., 1997), running (Lyngeraa et al., 2013)). For example, the hyperventilation‐induced hypocapnia attributed to lowering CBF at high exercise intensity during cycling (Hellstrom et al., 1996) is not observed during high‐intensity rowing (Faull et al., 2015), while both rowing and running display oscillatory patterns in CBF (Lyngeraa et al., 2013; Pott et al., 1997) that are not observed with cycling. Furthermore, the only study to date that has examined CBF patterns during running did not test a full range of intensities, only comparing responses at intensities of 50, 65, and 75% of heart rate reserve (Lyngeraa et al., 2013). Moreover, no single study has compared CBF responses between exercise modes, across a full range of exercise intensities. For this reason, the aim of the present study was to compare the impact of running and cycling on CBF [as indexed by transcranial Doppler (TCD) measures of middle cerebral artery blood velocity (MCAv)] across a full range of exercise intensities. In line with previous research, we hypothesized that exercise‐induced increases in MCAv for cycling would peak at 65% VO_2max_, while running would elicit a unique and markedly different profile compared to cycling due to the whole‐body nature of the exercise.

## 2 METHODS

### 2.1 Ethical approval

This study was approved by the Science, Technology, Engineering and Mathematics Ethical Review Committee, University of Birmingham (ERN_17‐1570) and adhered to the principles of the Declaration of Helsinki (2013). All volunteers provided written informed consent.

### 2.2 Participants

Twenty‐six physically active male (14) and female (12) (age 26 ± 8 years, height 171 ± 9 cm, body mass 69 ± 11 kg, running VO_2max_ 46.3 ± 6.3 mL/min/kg, cycling VO_2max_ 41.3 ± 7.8 mL/min/kg, see Table 1) volunteers took part in this study. Participants were accustomed to both running and cycling exercise, including running on a treadmill. Participants were not taking any medication (with the exception of oral contraception in females), were nonsmokers, and had no history or symptoms of cardiovascular, respiratory, or neurological disease. Participants were excluded if they self‐reported as sedentary (as defined by the Department of Health General Practise Physical Activity Questionnaire (2006)). Female participants could participate at any point during their menstrual cycle. All participants were instructed not to consume a large meal 4 hr before arrival (a light meal was permitted up to 2 hr prior and participants were advised to consume 0.5 L of water in the 4 hr preceding each exercise session), caffeine 6 hr before and alcohol 24 hr prior to the visit. Participants were also instructed to avoid vigorous exercise in the 24 hr prior to each experimental visit.

**TABLE 1:** Overall and subgroup baseline characteristics

Variable	Overall (*n* = 26)	Fit (*n* = 16)	Unfit (*n* = 7)
Sex (male: female)	14:12	10:6	2:5
Age (years)	26 ± 8	22 ± 5	26 ± 10
Body Mass (kg)	69.0 ± 10.9	67.4 ± 9.3	68.3 ± 6.9
Height (cm)	171 ± 9	172 ± 9	171 ± 10
Running VO_2max_ mL/min/kg	46.3 ± 6.3	53.4 ± 2.5	37.5 ± 1.9
Cycling VO_2max_ mL/min/kg	41.3 ± 7.8	45.3 ± 6.9	34.1 ± 3.4

Note

VO_2max_, maximal oxygen consumption. Values are mean ± *SD*.

### 2.3 Study design and protocol

A repeated‐measures, randomized crossover design was employed; requiring each participant to attend a total of four separate experimental exercise sessions. The first two exercise sessions involved the completion of modality‐specific VO_2max_ tests on a treadmill and bike ergometer, carried out in a randomized order. These tests began with a 5‐min graded warm‐up, whereby treadmill speed or resistance load was increased every minute until they rated a score of 11 on the Borg perceived exertion scale. Following the warm‐up participants were given the opportunity to stretch, before completing a standardized ramp protocol to determine VO_2max_. The standardized ramp protocol consisted of increased workload (treadmill speed/gradient or cycling ergometer resistance) in 3 min stages until VO_2max_ was reached. Participants were considered to have reached VO_2max_ if they achieved at least two of following criteria: respiratory exchange ratio > 1.1; a clear plateau in VO_2_ measures over a 30‐s period, or volitional fatigue. The remaining two exercise sessions involved two incremental exercise sessions; one running and one cycling, carried out in a randomized order. The incremental protocol involved running or cycling for 3‐min intervals at 35%, 50%, 65%, 80%, and 95% of VO_2max_ determined from the VO_2max_ test. All exercise sessions were preceded by a 5‐min warm‐up and succeeded by a 3‐min cool down.

### 2.4 Measurements

Bilateral middle cerebral artery velocity (MCAv) was measured as an index of CBF using TCD (Multi‐Dop X, DWL, Germany), with 2‐MHz probes placed above the zygomatic arch on the left and right side of the head. Probes were prepared with ultrasound gel and held in place with a headset (DiaMon®, DWL), with the neoprene strapping that held the face mask fitted over the top of the headset to help maintain this fixed position throughout the exercise. Search and identification procedures were performed in accordance with established guidelines (Willie et al., 2011) with settings kept constant between exercise sessions. The TCD filter was set at 350 for all sessions, as pilot testing established that the high‐frequency noise and electrical interference associated with heel strike during running was removed at this setting without affecting the waveform derived from the spectral trace. Beat‐to‐beat MCAv and HR measurements were recorded via an analog‐to‐digital converter (Powerlab 8/30, ADInstruments Ltd, New Zealand) and displayed in real time and stored for offline analysis using Lab Chart software (LabChart v7, ADInstruments Ltd). Time point markers were inserted during acquisition at the start of each increment during the protocol, with these markers used for temporal alignment with the respiratory data.

Oxygen consumption (VO_2_) and respiratory variables (ventilation rate, ventilation volume, partial pressure of end‐tidal CO_2_) were measured by indirect calorimetry (VyntusTM CPX, Carefusion, Germany), and the data recorded via Sentrysuite™ software (Version 2.19, Carefusion, Germany). These data were displayed in real time during testing and used to confirm each target intensity. Time point markers were inserted during data acquisition to allow for the temporal alignment with the data collected within Lab Chart software. During the last minute of each stage of all VO_2max_ and incremental protocols, participants rated perceived exertion (RPE) via the 15‐point Borg scale (Borg, 1982). Treadmill running was performed on a standard treadmill (Cosmos, Quaser, Germany) and cycling on a cycle ergometer (Sport Excalibur, Lode, The Netherlands).

### 2.5 Data analysis

Resting measures of mean MCAv (MCAv_mean_), P_ET_CO_2_, VO_2,_ and HR were averaged from the final 30 s of quiet seated rest, which was preceded by at least 10 min of sitting during equipment instrumentation and Doppler signal optimization. Exercising measures of these same variables were averaged over the final 30 s of each incremental stage. Systolic MCAv (sMCAv) and diastolic MCAv (dMCAv) data were obtained via the MCAv waveform trace (maximum and minimum values of the wave form, respectively), with pulsatility index (PI) calculated from these data (PI = sMCAv – dMCAv / MCAv_mean_). The MCAv measurements were obtained from the Doppler probe that provided the cleanest signal throughout the whole exercise session, which varied between participants but remained constant within participants across their two visits. MCAv data reported are presented as a change from rest.

In order to allow for assessment of the impact of fitness on the exercise‐induced cerebrovascular responses, data from treadmill VO_2max_ tests were used to categorize fitness level as either lower (*n* = 7; male: <40 mL/min/kg, female: <35 mL/min/kg) or higher fitness (*n* = 16; male: >45 mL/min/kg, female: >40 mL/min/kg). These fitness thresholds were based on ACSM guidelines (Riebe, Ehrman, Ligouri, & Magal, 2018).

### 2.6 Statistical analysis

Statistical analyses were performed using R (R Core Team, 2019 ; Singmann, Bolker, Westfall, Aust, & Ben‐Shachar, 2019). Factorial mixed‐model ANOVA were used to examine the within‐and‐between‐modality differences in the dependent variables of interest, alongside consideration of sex as a between‐subjects factor, with post hoc pairwise comparisons (Bonferroni corrected) used to identify the location of main and interaction effects present between modalities. Comparison of the exercise intensity response in relation to participant fitness was assessed using factorial mixed‐model ANOVA, with post hoc pairwise comparison (Bonferroni corrected) in the same manner as above. Data are presented as mean ± *SD*, comparisons as estimated marginal mean with 95% confidence intervals, with the threshold for statistical significance set at *p* < .05.

## 3 RESULTS

**FIGURE 1:**
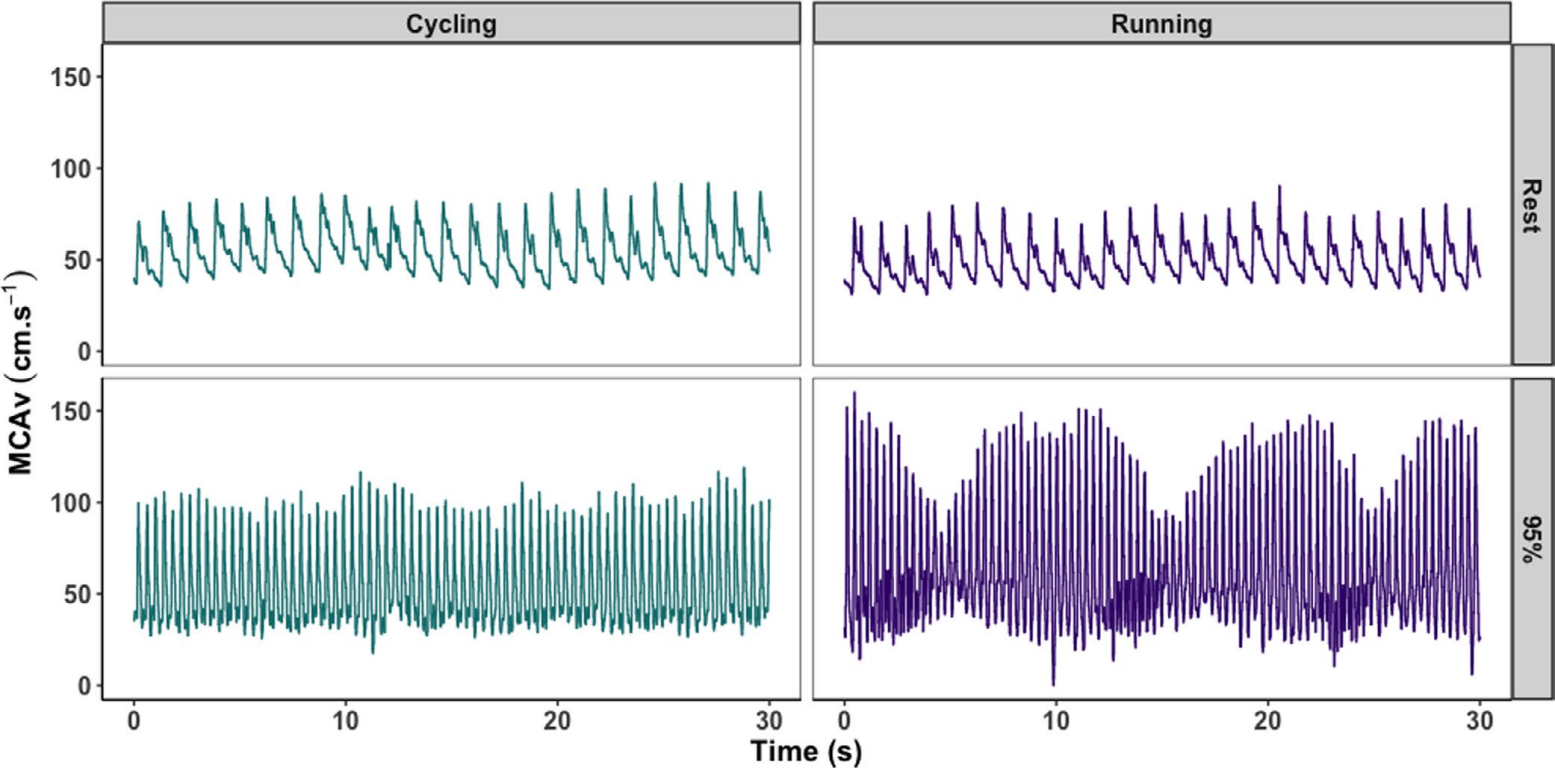
Representative beat‐to‐beat middle cerebral artery velocity (MCAv) profiles associated with cycling (left) and running (right), both at rest (top) and during exercise at 95% of VO_2max_. These data illustrate the stable beat‐to‐beat MCAv profile during cycling, compared to the oscillating profile seen in running. Data presented are taken from a single participant, across the full duration of the 30‐s averaging window

Twenty‐seven participants completed all four sessions, however, due to a poor Doppler signal quality in one participant during exercise trials only 26 participants were included in the statistical analysis. Table 2 shows the average resting baseline data across the two incremental exercise sessions for participants with full data sets, since there was no difference in resting baseline measures between these two sessions (all *p* > .05). Similarly, no significant main effects were seen between male and female participants for any primary outcome measure and as such all data were assessed and interpreted as a combined sex cohort (all *p* > .05). Figure 1 shows a representative trace of the beat‐to‐beat MCAv profiles during cycling and running exercise at rest and during exercise (at 95%VO_2max_).

**TABLE 2:** Resting baseline measures

Variable	Baseline (*n* = 26)
HR b/min	66 ± 11
MCAv (cm/s)	65 ± 13
sMCAv (cm/s)	98 ± 19
dMCAv (cm/s)	46 ± 9
PI (cm/s)	0.81 ± 0.11
PETCO_2_ (mm Hg)	33 ± 3

Note

dMCAv, diastolic middle cerebral artery blood velocity; HR, heart rate; MCAv, middle cerebral artery blood velocity; P_ET_CO_2_, Partial pressure of end‐tidal CO_2_; PI, pulsatility index; sMCAv, systolic middle cerebral artery blood velocity.

Values are mean ± *SD*.

### 3.1 Whole group comparison between running and cycling exercise

**FIGURE 2:**
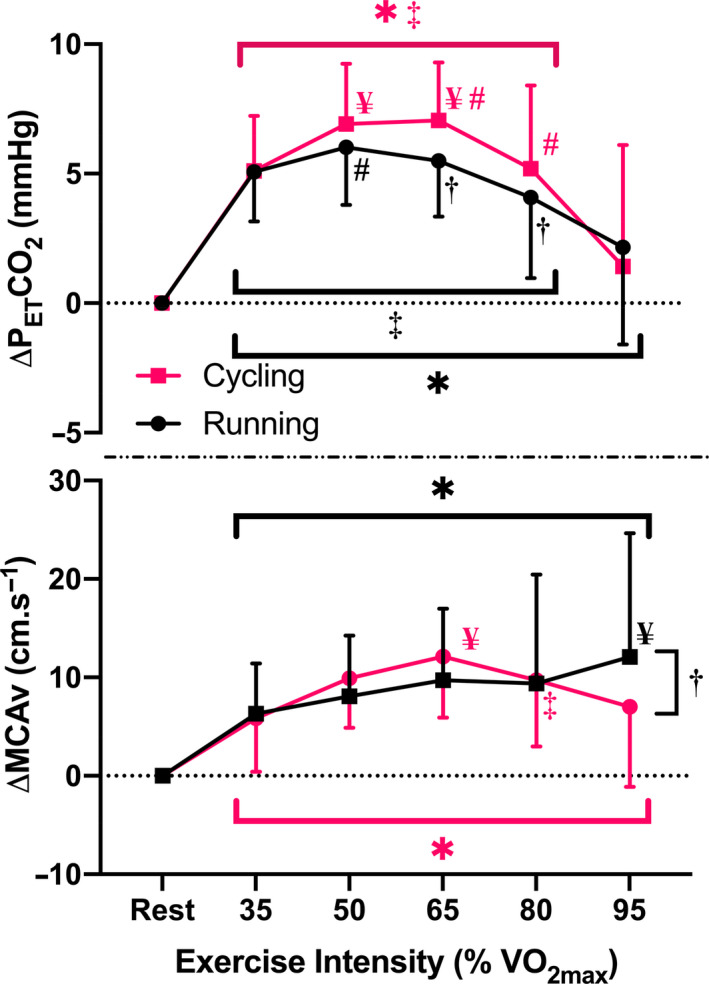
Comparison of change in partial pressure of end‐tidal carbon dioxide (P_ET_CO_2_) (top panel) and change in middle cerebral artery velocity (MCAv) from rest (bottom panel) during incremental running and cycling exercise (3‐min stages at 35%, 50%, 65%, 80%, 95% VO_2_max). Data are mean ± *SD* for 26 participants. *Significantly different from resting values (*p* < .05); ^¥^Significantly different from 35% VO_2max_ values (*p* < .05); ^#^Significantly different from 80% VO_2max_ values (*p* < .05); ^‡^Significantly different from 95% VO_2max_ values (*p* < .05); ^†^Significantly different between modalities (*p* < .05)

Mean MCAv increased from rest for both modalities during the incremental exercise protocol, although the pattern of change across the five intensities was different (interaction effect: *p* = .01, Figure 2). Post hoc analysis revealed that cycling MCAv_mean_ values peaked at 65% VO_2max_, significantly declining from this peak by the end of the protocol (65 vs. 95%, *p* < .01). However, MCAv_mean_ during running continued to increase from resting values across the full range of intensities, leading to a significant difference (*p* = .01) between the modalities at 95% VO_2max_ (change from rest: 12 ± 13 vs. 7 ± 8 cm/s for running vs. cycling).

In contrast, although there was significant interaction (*p* = .01) between running and cycling in measures of P_ET_CO_2_, an inverted‐U pattern in the data was seen in both modalities, significantly increasing from baseline at lower intensities before returning toward baseline values by 95% VO_2max_ (Figure 2). The interaction effect was mediated by an earlier peak during running (at 50% VO_2max_), which resulted in significantly lower P_ET_CO_2_ values at 65% and 80% VO_2max_ (both *p* < .05) compared to cycling. However, while significant, these differences were relatively small (1.6 mmHg and 1.1 mmHg, respectively) in comparison to the increase from baseline in either modality (i.e., all >4 mmHg). Similarly, while overall V_E_ increased significantly across the course of the exercise increments in both modalities (*p* < .01, Table 3), significant differences were seen between modalities at the same intensities as were seen in P_ET_CO_2_, with greater increases seen in running than in cycling at 50%, 65%, and 80% (*p* ≤ .03, Table 3).

**TABLE 3:** Measures of systolic and diastolic middle cerebral artery velocity and the calculated pulsatility index, and minute ventilation volume during incremental running and cycling

	Rest	35% VO_2max_	50% VO_2max_	65% VO_2max_	80% VO_2max_	95% VO_2max_
Running						
sMCAv (cm/s)	99.7 ± 18.3	121.5 ± 22.3* ^†^	133.0 ± 23.9* ^†^	144.5 ± 25.3* ^†^	139.2 ± 31.2* ^†^	141.4 ± 32.2 * ^†^
dMCAv (cm/s)	46.5 ± 9.8	44.2 ± 11.3	40.5 ± 12.4* ^†^	33.2 ± 12.5* ^†^	34.9 ± 12.0* ^†^	37.3 ± 13.4* ^†^
PI (cm/s)	0.82 ± 0.10	1.09 ± 0.16* ^†^	1.28 ± 0.27* ^†^	1.51 ± 0.28* ^†^	1.41 ± 0.23* ^†^	1.36 ± 0.26* ^†^
V_E_ (L/min)	10.2 ± 2.9*	26.4 ± 4.8*	40.0 ± 7.1* ^†^	57.2 ± 11.2* ^†^	77.3 ± 15.3* ^†^	99.6 ± 20.5*
Cycling						
sMCAv (cm/s)	97.7 ± 19.2	113.1 ± 20.7* ^†^	124.3 ± 21.3* ^†^	130.1 ± 21.4* ^†^	127.3 ± 20.4* ^†^	121.7 ± 21.2* ^†^
dMCAv (cm/s)	45.8 ± 8.6	46.2 ± 9.8	48.2 ± 9.8^†^	49.0 ± 10.0^†^	46.7 ± 10.4^†^	44.2 ± 11.9^†^
PI (cm/s)	0.81 ± 0.11	0.96 ± 0.15* ^†^	1.03 ± 0.13* ^†^	1.07 ± 0.12* ^†^	1.10 ± 0.16* ^†^	1.11 ± 0.22* ^†^
V_E_ (L/min)	9.9 ± 4.6*	24.8 ± 4.7*	36.5 ± 7.5* ^†^	51.2 ± 9.5* ^†^	69.6 ± 13.0* ^†^	96.5 ± 22.1*

Note

Data are mean ± *SD* for 26 participants.

dMCAv, diastolic middle cerebral artery velocity; PI, pulsatility index; sMCAv, systolic middle cerebral artery velocity; V_E_, Minute ventilation.

* Significantly different from resting values (*p* < .05).

^†^ Significantly different between modalities (*p* < .05).

Table 3 displays sMCAv, dMCAv, and PI data for both modalities across the full range of intensities tested. For all three parameters there was a significant interaction effect between modalities across the five exercise intensities (all *p* < .01). Post hoc analysis revealed that sMCAv was ~8–20 cm/s higher during running than cycling for all exercise intensities (all *p* < .05), while dMCAv was ~6–15 cm/s lower during running than cycling at 50%, 65%, and 80% VO_2max_ (all *p* < .05). Correspondingly, PI was greater during running than cycling for all exercise intensities (all *p* ≤ .01, Table 3).

### 3.2 Fitness‐dependent variation in running response

**FIGURE 3:**
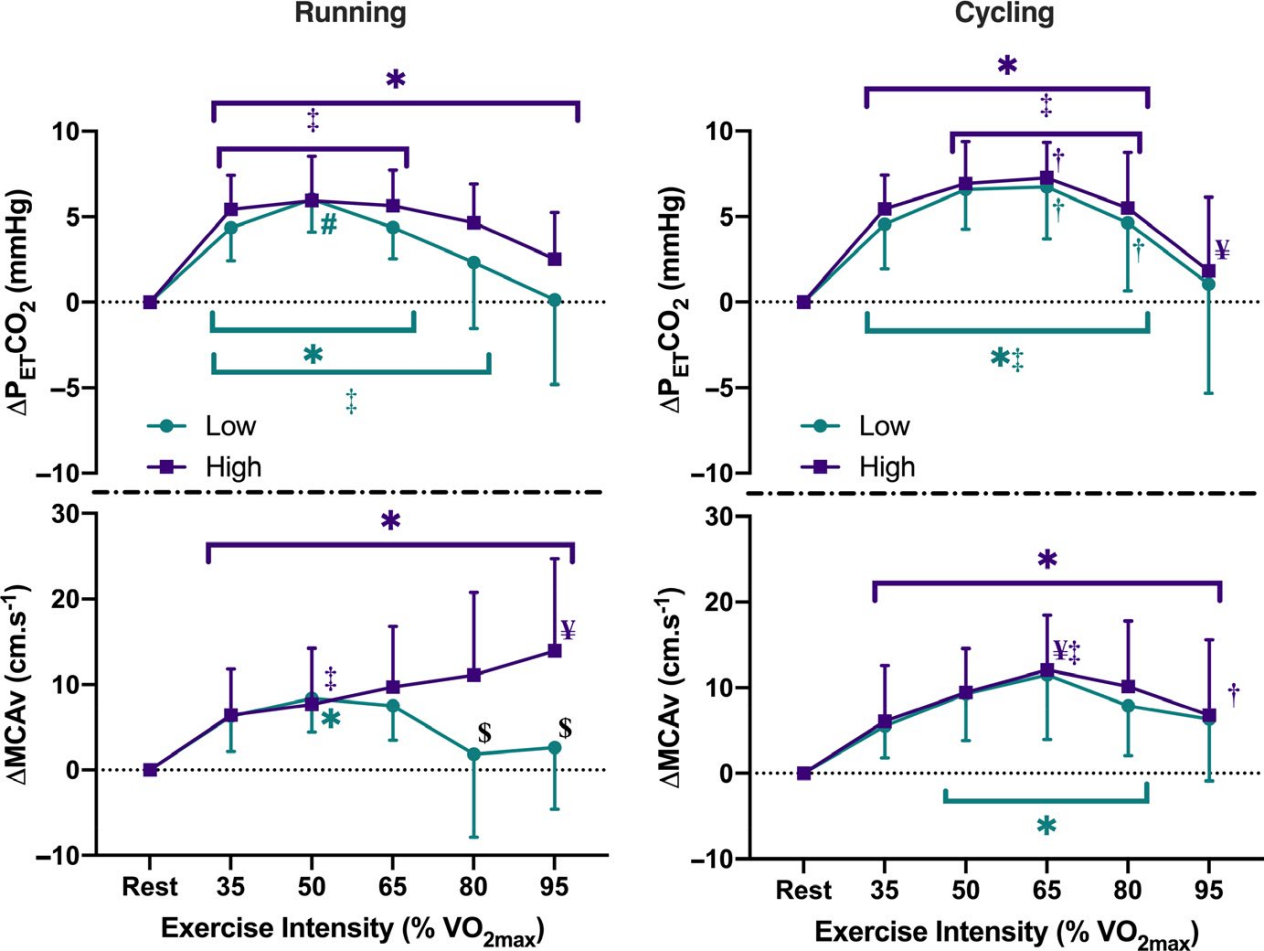
Comparison of change in partial pressure of end‐tidal carbon dioxide (P_ET_CO_2_) (top panels) and change in middle cerebral artery velocity (MCAv) from rest (bottom panels) during incremental running (left) and cycling (right) exercise (3‐min stages at 35%, 50%, 65%, 80%, 95% VO_2_max), between participants characterized as high (male: >45 mL/min/kg; female: >40 mL/min/kg) or low fitness (male: <40 mL/min/kg; female: <35 mL/min/kg). ^*^Significantly different from resting values (*p* < .05); ^¥^Significantly different from 35% VO_2max_ values (*p* < .05); ^#^Significantly different from 80% VO_2max_ values (*p* < .05); ^‡^Significantly different from 95% VO_2max_ values (*p* < .05); ^$^Low fitness significantly different from high fitness (*p* < .05); ^†^Significantly different between modalities (*p* < .05)

Analysis of MCAv_mean_ responses in relation to participant fitness showed a significant interaction between fitness level and the MCAv_mean_ response to incremental exercise (*p* = .04, see Figure 3). Specifically, while an inverted‐U pattern for the MCAv_mean_ response was similar between fitness groups for incremental cycling, differences between the two fitness groups were observed at intensities above 65% VO_2max_ when running. While MCAv_mean_ continued to increase in the high fitness group as intensity increased, MCAv_mean_ in the low fitness group decreased, with blood velocity 9 cm/s (95% CI [3, 15]; *p* = .01) and 11 cm/s (95% CI [5, 17]; *p* < .01) greater in the high fitness group at 80% and 95%, respectively. The continued elevation in the high fitness subgroup also resulted in a significantly higher MCAv_mean_ at 95% VO_2max_ in comparison to the same intensity during cycling for this group (14 ± 11 vs. 7 ± 9 cm/s, respectively, *p* < .01).

As with the whole group observations, an inverted‐U pattern was seen in both modalities for P_ET_CO_2_. Comparison of responses between the two subgroups during running showed no significant differences between them, with both exhibiting similar patterns in response to that seen in cycling (three‐way interaction effect: *p* = .52; Figure 3). There were significant differences between modalities at 65% VO_2max_ for both fitness subgroups, and at 80% VO_2max_ in the low fitness subgroup alone (all *p* < .05).

## 4 DISCUSSION

The primary purpose of this study was to complete the first formal comparison of CBF responses (indexed via MCAv changes) to running and cycling across a full range of exercise intensities, within the same participant cohort using a stepped, incremental protocol. Our main findings were: a) consistent with our hypothesis, MCAv_mean_ and P_ET_CO_2_ during cycling both peaked at 65% VO_2max_ before decreasing back toward resting values at the higher intensities; b) in contrast, MCAv_mean_ during running continued to increase up to 95% VO_2max_ despite a relative decrease in P_ET_CO_2_ above 65% VO_2max_, although the MCAv_mean_ responses appeared divergent within the whole cohort tested; (c) subsequent subgroup analysis revealed that increases in MCAv_mean_ above 65% VO_2max_ in running were observed in participants with higher aerobic fitness levels only (male: >45 mL/min/kg; female: >40 mL/min/kg), whereas the exercise‐induced MCAv_mean_ response in participants of lower fitness followed a biphasic response similar to that seen in cycling (for fit and unfit groups), and (d) the beat‐to‐beat MCAv profile associated with running displayed rhythmic oscillations that were not observed during cycling. Taken together, these findings demonstrate that exercise‐induced increases in MCAv_mean_ during incremental running and cycling differ at higher exercise intensities, with the pattern of the CBF response during running for fitter individuals dissociating from the regulatory influence of PCO_2_ at near maximal intensity. Thus, modality‐specific differences in beat‐to‐beat flow patterns may alter CBF regulation processes and affect the complex integration of factors regulating CBF during exercise. Ultimately, this may lead to modality‐specific differences in vascular adaptation processes linked to exercise (e.g., shear stress) and therefore influence how different types of exercise may affect brain vascular health.

### 4.1 Whole group comparison between running and cycling exercise

A key novel finding from the present study was the different pattern of exercise‐induced increases in MCAv_mean_ between running and cycling during incremental exercise. Contrary to the biphasic relation typically reported from cycling‐based studies (reviewed in (Smith & Ainslie, 2017)), we observed that MCAv_mean_ recorded during running increased linearly up to 95% VO_2max_. Furthermore, this linear increase in MCAv_mean_ occurred despite a biphasic P_ET_CO_2_ response during incremental running, similar to that produced with cycling (see Figure 2). Collectively, this indicates an apparent uncoupling of the regulatory influence of PCO_2_ on MCAv at higher running intensities, which is similar to that reported during high‐intensity (i.e., 30‐s all out) rowing (Faull et al., 2015). As such, the assumption that exercise‐induced changes in CBF are the same for all exercise modalities is not correct.

One possible explanation for the observed dissociation between P_ET_CO_2_ and MCAv_mean_ during high‐intensity running may be related to running‐induced oscillations in the beat‐to‐beat MCAv profile (Figure 1). Running‐induced MCAv oscillations have been previously observed by Lyngeraa and colleagues (Lyngeraa et al., 2013). They demonstrated that pulse pressure oscillations induced via the summation of pressure waves originating in the arterial tree and in response to heel strike (i.e., the beat phenomenon (Palatini et al., 1989)), were transferred to the cerebrovasculature while running. However, Lyngeraa and colleagues did not examine the effect of near maximal intensities on this response (their maximum intensity was 75% of heart rate reserve) and did not assess how MCAv changed relative to the key CBF regulator, PCO_2_ (as we have done via measures of P_ET_CO_2_). Unfortunately we were unable to reliably record blood pressure during the running exercise, however, one would expect blood pressure to increase with increasing exercise intensity, in line with the exercise pressor response (Smith, Mitchell, & Garry, 2006). This, combined with the beat‐to‐beat MCAv oscillations, may have challenged cerebral autoregulation sufficiently to increase MCAv_mean_ at 80% and 95% VO_2max_ despite the relative hypocapnia. Interestingly, attenuated dynamic cerebral autoregulation has been reported in fitter individuals (Drapeau et al., 2019; Labrecque et al., 2017; Lind‐Holst et al., 2011), which may explain the observed fitness effect related to this modality‐specific increase in MCAv_mean_ observed at higher intensities (discussed more below). Further research exploring how these oscillatory patterns during running are linked to the regulatory control and long‐term vascular adaptation is needed. Furthermore, while not investigated within the current study, the potential role of elevated sympathetic activity in controlling cerebrovascular responses to exercise and the interplay between the sympathetic nervous system and changes in blood pressure may be relevant for the differences observed here (see review by: Brassard, Tymko, & Ainslie, 2017). The complexity of the role that sympathetic control plays and its potential interaction with both modality‐ and intensity‐dependent changes in exercise responses warrants further study, specifically focussed on the measurement of sympathetic activity.

### 4.2 Impact of physical fitness on running response to incremental exercise

Although we showed a statistically significant effect between exercise‐induced changes in MCAv_mean_ between our incremental running and cycling protocols, we also observed a greater deviation around the mean response during running compared to cycling (see Figure 2). Anecdotally it seemed that participants’ cardiorespiratory fitness levels may have mediated this variation. To explore this potential interaction, two subgroups were investigated within our cohort to explore a potential fitness effect (i.e., VO_2max_ > 45 mL/min/kg vs. <40 mL/min/kg in males; and >40 mL/min/kg vs. <35 mL/min/kg in females). These data indicated that participants with higher fitness produced steeper increases in MCAv_mean_ during the 80% and 95% VO_2max_ exercise intensities in comparison to less fit participants, whereas a more homogenous response across all participants was observed for cycling. Interestingly, despite a clear MCAv_mean_ response pattern difference between the two subgroups when running, similar differences were not seen in measures of P_ET_CO_2_, with both fitness subgroups exhibiting a significant decline above 65% VO_2max_ and similar patterns of response in both running and cycling. Additionally, while significant differences were seen in V_E_ between modalities, these changes were in line with the slight differences in P_ET_CO_2_ at the same intensities and do not demonstrate the level of divergence needed for changes in ventilation to play a significant role in modality‐specific responses. These observations provide a rationale to further explore how greater levels of fitness/training may influence this running‐specific MCAv response, with differences in running style or efficiency, and/or breathing rate potentially mediating an altered response for fitter individuals.

However, as mentioned above, the impact of fitness on cerebrovascular regulatory control may explain this effect. As well as attenuated dynamic cerebral autoregulation (Drapeau et al., 2019; Labrecque et al., 2017), fitter individuals have been reported to have lower cerebrovascular reactivity to CO_2_ (Thomas et al., 2013), although this is not a universal finding (Bailey et al., 2013; Foster, Steventon, Helme, Tomassini, & Wise, 2020), meaning that relative hypocapnia may induce less vasoconstriction in these fitter individuals; although it is not obvious why this would be any different between running and cycling modalities. Interestingly, Zhou and colleagues have shown that stroke volume in elite runners increases continuously with increasing running intensities (up to maximal) during treadmill exercise (Zhou et al., 2001). Thus, given the regulatory contribution of cardiac output to CBF, a greater stroke volume (and therefore cardiac output) may also help explain the linear relationship between MCAv_mean_ and running intensity in fitter individuals. Overall, the combination of modality‐specific responses (e.g., pulse pressure oscillations, greater stroke volume) together with fitness‐induced adaptative alterations in cerebral blood flow regulation may produce a situation where the cerebrovasculature is more pressure passive for fitter individuals when running at high intensities. While little research has been conducted into the safety of high‐intensity exercise in well‐trained individuals, as recently highlighted with regard to interval‐based high‐intensity exercise, the potential risk that changes (especially rapid and large) in blood pressure may have in the cerebrovasculature if not properly regulated need to be considered (Calverley et al., 2020).

### 4.3 Limitations

Transcranial Doppler (TCD) ultrasound was used to assess the CBF response to exercise in this study. While it is well recognized that TCD only quantifies the velocity of blood within the insonated artery rather than flow per se (Willie et al., 2011), obtaining whole‐body, ecologically valid exercising CBF data is not possible via other approaches that address the TCD velocity‐based limitation (Tymko, Ainslie, & Smith, 2018) (e.g., duplex Doppler or ASL‐MRI). To ensure we interpreted our findings within the context of the measurement (Ainslie & Hoiland, 2014), we followed established standardized procedures to limit any measurement bias (Willie et al., 2011), and report change in MCAv from the session specific resting value rather than absolute values when comparing the modality and exercise intensity effects. Recent studies have demonstrated that the diameter of intra‐cranial arteries, such as the MCA, are subject to change in response to exercise and PaCO_2_ (Coverdale, Badrov, & Shoemaker, 2017; Coverdale, Gati, Opalevych, Perrotta, & Shoemaker, 2014; Coverdale, Lalande, Perrotta, & Shoemaker, 2015; Verbree, Bronzwaer, & van Buchem, 2017; Verbree et al., 2014). As such, the increases in MCAv observed during the high‐intensity incremental running protocol may have been influenced by this, however, it seems unlikely that this would only apply here and not during the other high‐intensity exercise measures of MCAv, where a diameter change (i.e., reduction via hypocapnic‐induced vasoconstriction) would only underestimate the true effect on CBF.

We also used P_ET_CO_2_ as an index of PaCO_2_ to assess the PaCO_2_‐CBF relation. At rest, it has been shown that P_ET_CO_2_ accurately represents PaCO_2_ (Williams & Babb, 1997), however, during exercise P_ET_CO_2_ may overestimate PaCO_2_ (Benallal & Busso, 2000; Williams & Babb, 1997). Moreover, differences in breathing patterns between exercise modalities may further influence this relation between P_ET_CO_2_ and PaCO_2_ during exercise. Indeed, ventilatory patterns are different between running and cycling for maximal exercise (Tanner, Duke, & Stager, 2014), with tidal volume larger for cycling. Therefore, running may result in greater blood (and tissue) retention of PCO_2_, and may be one potential explanation for the differential blood flow pattern we saw between running and cycling. However, it is not obvious why this would be differentially affected by fitness level between modalities, or why fitness‐specific differences in MCAv are seen during incremental exercise in running but not cycling, despite similar patterns in end‐tidal CO_2_ across all conditions.

The inclusion of female participants within the current cohort at any stage in their menstrual cycle and regardless of oral contraceptive usage is also a potential limitation, due to known variations in cerebrovascular function dependent on changes in circulating sex hormone levels (Krause, Duckles, & Pelligrino, 2006). While this does limit our capacity to interpret how sex hormones may alter the responses seen within our data, this decision was made to maximize inclusion and the order of visits was randomized in order to reduce the likelihood that findings were affected by female participants within a single menstrual cycle phase. Although alternate strategies such as inclusion within a particular menstrual phase have previously been utilized to control for sex hormone variations, these approaches limit our understanding to a single phase and are problematic due to high inter‐individual variability in sex hormone profiles across the cycle and the length of said cycles (Häggström, 2014; Sims & Heather, 2018). The influence of sex hormones on cerebrovascular function within the context of exercise and modality warrants further investigation, with the influence of sex hormones at multiple stages of the menstrual cycle and with and without the use of oral contraceptives.

### 4.4 Perspective

This study was the first to formally compare the CBF profiles of running and cycling across a full range of exercise intensities. We demonstrated that running and cycling have unique profiles for MCAv_mean_ at exercise intensities exceeding 65% VO_2max_ during a stepped incremental protocol, with the regulatory influence of P_ET_CO_2_ uncoupling from running‐induced MCAv_mean_ responses at the near maximal stage. This running‐specific elevation in MCAv_mean_ at higher intensities appears to be dependent on participant fitness, with lower fitness participants showing response profiles more akin to those seen in cycling. To confirm whether elevations in MCAv translate to elevated shear stress and cyclic strain stimuli with the capability of driving unique cerebrovascular adaptation between exercise modalities, future studies are required to quantify the magnitude of adaptive signaling (e.g., NO) and ultimately the functional change in brain vascular structure and function with repeated exposure (i.e., training). Such knowledge will help determine the most effective exercise strategies that enhance positive cerebrovascular adaptation and optimize brain health for both healthy and clinical populations.

## CONFLICT OF INTEREST

The authors declare that there is no conflict of interest.

## AUTHOR CONTRIBUTIONS

RAIL and SJEL were involved in conception and design. RJF, RS, CVB, GMI, RAIL, and SJEL were involved in data collection. RJF and RS were involved in data extraction. RJF, SRW, and SJEL were involved in data analysis. RJF, SRW, and SJEL were involved in statistical analysis, figure preparation, and draft of manuscript. RJF, SRW, RS, CVB, GMI, RAIL, and SJEL were involved in revision, critique, and final approval of the manuscript.
